# Muscarinic acetylcholine receptor 1 gene polymorphisms associated with high myopia

**Published:** 2009-09-04

**Authors:** Hui-Ju Lin, Lei Wan, Yuhsin Tsai, Wen-Chi Chen, Shih-Wei Tsai, Fuu-Jen Tsai

**Affiliations:** 1 Department of Ophthalmology, China Medical University Hospital, Taichung, Taiwan; 2 Department of Medical Genetics, China Medical University Hospital, Taichung, Taiwan; 3 School of Chinese Medicine, College of Chinese Medicine, China Medical University, Taichung, Taiwan; 4 Department of Biotechnology, Asia University, Taichung, Taiwan; 5 Graduate Institute of Integrated Medicine, College of Chinese Medicine, China Medical University, Taichung, Taiwan; 6 Institute of Environmental Health, College of Public Health, National Taiwan University, Taipei, Taiwan; Correspondence to: Fuu-Jen Tsai, M.D., Ph.D., Department of Medical Genetics, China Medical University Hospital, No. 2 Yuh Der Road, Taichung 404, Taiwan; Phone: 886-4-22052121 ext. 2041; FAX: 886-4-22033295; email: d0704@mail.cmuh.org.tw

## Abstract

**Purpose:**

Numerous studies, including those using animal models of myopia development and human clinical trials, have shown that the non-selective muscarinic antagonist atropine is effective in preventing the axial elongation that leads to myopia development. Among all of the muscarinic acetylcholine receptors (mAChRs), mAChR 1 (M1) was the most effective in preventing myopic eye change. Our specific aim in this study was to examine the association between high myopia and polymorphisms within the muscarinic acetylcholine receptors 1 gene* (CHRM1)*.

**Methods:**

The participants comprised of a high myopia group (n=194; age range, 17–24 years) having a myopic spherical equivalent greater than 6.5 diopters (D) and a control group (n=109; age range, 17–25 years) having a myopic spherical equivalent less than 0.5 D. Genotyping was performed using an assay-on-demand allelic discrimination assay. Polymerase chain reaction (PCR) was performed using 96 well plates on a thermal cycler. The polymorphisms detected were S1 (*CHRM1* rs11823728), S2 (*CHRM1* rs544978), S3 (*CHRM1* rs2186410), and S4 (*CHRM1* rs542269).

**Results:**

There was a significant difference in the distribution of S2 and S4 between the high myopia and control groups (p=2.40×10^−6^ and 2.38×10^-8^, respectively). The odds ratios of AA genotype of S2 and GG genotype of S4 were both 0.08 (95% confidence interval [CI]: 0.02–0.29 and 0.02–0.36, respectively). Logistic regression test revealed S1, S2, and S4 *CHRM1* as all being significant in the development of high myopia. Moreover, the distributions of haplotype 4 (Ht4; C/A/A/A) differed significantly between the two groups (p=3.4×10^−5^, odds ratio: 0.1, 95% CI: 0.03–0.34).

**Conclusions:**

Our results suggest that the S2 and S4 polymorphisms of *CHRM1* are associated with susceptibility for developing high myopia. S1, S2, and S4 *CHRM1* had a co-operative association with high myopia.

## Introduction

Clinically significant refractive errors are the most common type of visual disorders. Myopia affects more than half of the young adult population in the world [1-3]. In Asian countries, the prevalence of myopia has approached epidemic proportions [4]. A range of animal models of myopia development have demonstrated that the non-selective muscarinic acetylcholine antagonist, atropine, is effective in preventing the axial elongation that leads to myopia development [5-9]. Human clinical trials have also shown an effectiveness of daily atropine administration in reducing the progression of myopia. The muscarinic acetylcholine receptors (mAChRs) are a group of neurotransmitter proteins belonging to the seven transmembrane superfamily of receptors. Five distinct receptor genes (*CHRM1*-*CHRM5*) have been cloned, each encoding a muscarinic receptor protein 1–5 (M1-M5) with specific pharmacological properties [10-12]. In the study of guinea pigs, M1 and M4 subtypes were found to be significantly increased in the posterior sclera of form-deprived myopic eyes. Studies of the individual muscarinic antagonists had revealed that M1 selective pirenzepine and M2/M4 selective himbacine were effective in inhibiting the development of myopia [8,13,14]. Pirenzepine, an M1 selective antagonist, is effective in preventing the progression of myopia in a dose-dependent manner in both mammalian and avian models [8,13,15]. According to the previous studies, we understood that among all mAChRs, M1, M2, and M4 might have the most important roles in the “stop” signal of myopic progression. In vitro studies had found that mAChR antagonists inhibit proliferation and matrix synthesis of the cartilaginous sclera. There was a rank order of the selective antagonists in the function of reducing incorporation of sulfate into glycosaminoglycans (GAGs) by chondrocytes. The rank of potency was M1 (pirenzepine-telenzepine) > Ml and M3 (4-diphenylacetoxy-N-methylpiperidine methiodide [4-DAMP]) > M2 (gallamine). The ranking suggests that the Ml subtype is the most dominant type to regulate scleral growth [16].

Many studies have suggested that myopia is a complex disease with multiple causes including the interaction of multiple genes with environmental stimuli [17]. Therefore, to understand myopia, it is necessary to take both genes and environment into account [17]. There is evidence supporting the premise that myopia and refractive errors are in large part genetically determined. This comes from twin studies and from studies of refractive errors in parents and their children [18,19]. A well conducted study has shown that refractive errors were much more strongly correlated in monozygotic twins than in dizygotic twins [19]. In the present study, we investigated the association of genetic polymorphisms in M1 and high myopia. The gene encoding the M1 receptor, muscarinic acetylcholine receptors 1 gene* (CHRM1)*, is localized to 11q13. Four genetic polymorphisms were selected using HapMap, genotypes were analyzed within Haploview and tag SNPs were selected using the Tagger function. The polymorphisms were rs11823728 in the 3′UTR and rs544978, rs2186410, and rs542269 in the intron.

## Methods

### Patients

Between February to November 2004, we measured the refractive error in 3,000 volunteers. All of the participants were medical students, unrelated, and Taiwan-born Han Chinese. The enrolled participants in this study were aged 16–25 years (mean age of 18±3.2 years), and the male to female ratio was 1.8:1.0. All participants had a visual acuity with distance correction of 0.2 logMAR (20/32) or better. Refractive error was measured in diopters (D) and determined by the mean spherical equivalent (SE) of the two eyes of each individual after administering one drop of cycloplegic drug (1% mydriacyl; Alcon, Berlin, Germany). Individuals with myopia greater than or equal to 6.5 D (both eyes) were included in the high myopia group, and those with myopia less than 0.5 D and hyperopia less than 1.0 D (both eyes) were included in the control group. Patients with astigmatism with a refractive error more negative than -0.75 D were excluded from the study since it would alter the results of SE. The study was reviewed by the ethics committee of the China Medical University Hospital (Taichung, Taiwan) and performed in accordance with the tenets of the Declaration of Helsinki for research involving human subjects. Informed consent was obtained from all participants. A comprehensive ophthalmic examination and blood collection were performed. None of the participants had ocular disease or insult such as retinopathy, prematurity, neonatal problems, a history of genetic disease, and connective tissue disorders associated with myopia such as Strickler or Marfan syndromes. Clinical examinations included visual acuity, refraction error, slit-lamp examination, ocular movements, intraocular pressure, and fundoscopy. Patients with organic eye disease, a history or evidence of intraocular surgery, history of cataract, glaucoma, retinal disorders, or laser treatment were excluded. As with all data collection procedures, auto-refraction (Auto-refractor/auto-keratometer [ARK 700A; Nikon, Tokyo, Japan]) was conducted on both eyes by experienced optometrists who were trained and certified on the study protocols. Refractive data, sphere (s), negative cylinder ©, and axis measurements were analyzed by calculating SEM refractive error.

### Genotype determinations

To select the most representative SNPs to capture the majority genetic variation, SNP genotype information was downloaded in December, 2007 from the HapMap (haplotype map of the human genome project) HCB (Han Chinese in Beijing, China) or JPN (Japanese) population from an extended 30Kb region of Chromosome 11. HapMap genotypes were analyzed within Haploview and Tag SNPs were selected using the Tagger function. Four tag SNPs were selected for each gene with r^2^≥0.80 to capture 80% of genotype information in the region. The average tag SNP was with r^2^=0.901. Genotyping was achieved using an assay-on-demand allelic discrimination assay and a detection system according to the manufacturer’s instructions (Applied Biosystems Co, Foster City, CA). The polymerase chain reaction (PCR) reaction contained 10 ng of genomic DNA, 10 μl TaqMan master mix, and 0.125 μl of 40X assay mix. PCR was performed using 96 well plates on a thermal cycler (ABI 9700; Applied Biosystems). Reaction conditions were 50 °C for 2 min and 95 °C for 10 min followed by 40 cycles of 95 °C for 15 s and 60 °C for 1 min. Four polymorphisms met the criteria and were selected in this study. They were S1 (*CHRM1* rs11823728), S2 (*CHRM1* rs544978), S3 (*CHRM1* rs2186410), and S4 (*CHRM1*, rs542269). Haplotypes were inferred from unphased genotype data using the Bayesian statistical method available in the software program, Phase 2.1. All five SNPs were analyzed with the Phase 2.1 software [20].

### Statistical analysis

Genotypes were obtained by direct counting with subsequent calculation of allele frequencies. Data were analyzed using the χ^2^ test or Fisher's exact test, and p values were calculated using the Minitab program (Minitab Inc., San Jose,CA). A p value less than 0.05 was considered statistically significant. The odds ratios (OR) and the corresponding 95% confidence intervals (CI) were calculated for associations concerning allele and genotype. Bonferroni corrections for multiple comparisons were performed [21]. All of the detected SNPs were assessed for Hardy–Weinberg equilibrium (HWE) using the χ^2^ test [22]. Stepwise logistic regression was used to determine if any one accounted for the effects of others. This was performed using the STATA package (version 8.2; Stata Corp., College Station, TX).

## Results

Volunteers enrolled in this study were on the basis of the following data: age, 16–25 years (mean, 18±3.3 years); male-to-female ratio, 1.48:1.0; mean axial length (AXL), 24.8 mm; and mean spherical equivalent (SE), –4.5D. There were no significant difference between the control and cases groups with respect to age, gender, cornea diopter (CD), anterior chamber depth (ACD), and LT (lens thickness; Table 1). The study group comprised of 194 patients with high myopia, and the control group consisted of 109 individuals with normal eyes. Four polymorphisms selected for this study are as described in Table 2: S1 (*CHRM1* rs11823728), S2 (*CHRM1* rs544978), S3 (*CHRM1* rs2186410), and S4 (*CHRM1* rs542269). The genotype distributions of the *CHRM1* polymorphism for both the high myopia group and controls are shown in Table 2. There was significant difference in genotype and allele distribution of the S2 (*CHRM1* rs544978)**polymorphism between high myopia patients and normal controls (*p*=2.40×10^-6^ and 0.004, respectively) after Bonferroni correction (Table 2 and Table 3). For S2 (*CHRM1* rs544978), the genotype frequencies of G/G, A/G, and A/A were 74.74%, 23.71%, and 1.55%, respectively, in the high myopia group and 69.72%, 12.84%, and 17.43%, respectively, in the control group (Table 3). The allelic frequency of G and A was 86.60% and13.40%, respectively, in the high myopia group and 76.15% and 23.85%, respectively, in the control group (Table 2). There was also a significant difference in the genotype distribution of the S4 (*CHRM1* rs542269) polymorphism (p=2.38×10^−8^ after Bonferroni correction) with the genotype frequencies of A/A, A/G, and G/G being 76.80%, 22.16%, and 1.03%, respectively, in the high myopia group and 83.49%, 2.75%, and 13.76%, respectively, in the control group. The odds ratios for an association with the S4 genotype were 8.75 (95% CI: 2.64–29.04) for A/G and 0.08 (95% CI: 0.02–0.36) for G/G. However, there were no significant differences in the distribution of S4 alleles between the high myopia and control groups (p=1.164 after Bonferroni correction; Table 2). The frequencies of S4 alleles A and G were 87.89% and 12.11%, respectively, in the high myopia group and 84.86% and 15.14%, respectively, in the control group (Table 2). For S1 (*CHRM1* rs11823728), the frequencies of genotypes G/G, A/G, and A/A were 96.39%, 3.09%, and 0.52%, respectively, in the high myopia group and 97.25%, 2.75%, and 0%, respectively, in the control group. The frequencies of the G and A alleles were 97.94% and 2.06%, respectively, in the high myopia group and 98.62% and 1.38%, respectively, in the control group. There were no significant differences in the genotype and allele distributions of the S1 (*CHRM1* rs11823728) polymorphism between the high myopia and control groups (p=2.96 and 2.176, respectively; Table 2 and Table 3). There were also no significant differences in the genotype distributions or allele frequencies of the S3 polymorphism (*CHRM1* rs2186410) between the two groups. The p values of S3 (*CHRM1* rs2186410) genotype and allele were p=0.12 and 3.98, respectively (Table 2 and Table 3).

**Table 1:** Characteristics of the study subjects and the partial correlation between spherical equivalent and other ocular components.

**Characteristics**	**Control (SD) n=109**	**Cases (SD) n=194**	**All subjects (SD) n=303**
Age, mean(SD), years	18 (2.8)	18 (3.5)	18 (3.3)
Female, n (%)	44 (40.4%)	78 (40.2%)	122 (40.3%)
SE, mean (SD), D	0.03 (0.31)	−8.82 (2.4)	−4.5 (2.1)
AXL, mean (SD), mm	23.8 (0.78)	26.8 (1.8)	24.8 (2.5)
CD, mean (SD), D	43.7 (0.8)	44.2 (1.8)	43.9 (1.2)
ACD, mean (SD), mm	3.57 (0.24)	3.89 (0.32)	3.62 (0.4)
LT, mean (SD), mm	3.8 (0.64)	4.0 (0.59)	3.9 (0.6)

SE: spherical equivalent (spherical equivalent equal to the sphere plus 1/2 the cylinder) spherical equivalent, AXL: axial length, CD: cornea diopter, ACD: anterior chamber depth, LT: lens thickness.

**Table 2:** Association between genotype distributions of *CHRM1* polymorphisms and individuals with high myopia.

**Polymorphisms**	**Individuals with myopia > 6.0D (%)**	**Individuals with myopia < 0.5D (%)**	**OR**	95% CI*	**p value^#^/Cp-value^##^**
rs11823728 (S1)					
G/G	187 (96.3)	106 (97.2)	1	-	0.74/2.96
A/G	6 (3.1)	3 (2.8)	1.13	0.28–4.63	
A/A	1 (0.5)	0 (0)	-		
rs544978 (S2)					
G/G	145 (74.7)	76 (69.7)	1	-	5.99×10^−7^/2.40× 10^−6^
A/G	46 (23.7)	14 (12.8)	1.72	0.89–3.33	
A/A	3 (1.5)	19 (17.4)	0.08	0.02–0.29	
rs2186410 (S3)					
A/A	150 (77.3)	89 (81.7)	1	-	0.03/0.12
A/G	24 (12.3)	4 (3.7)	3.56	1.2–10.59	
G/G	20 (10.3)	16 (14.7)	0.74	0.37–1.51	
rs542269 (S4)					
A/A	149 (76.8)	91 (83.5)	1	-	5.96×10^−9^/2.38×10^−8^
A/G	43 (22.2)	3 (2.8)	8.75	2.64–29.04	
G/G	2 (1.0)	15 (13.8)	0.08	0.02–0.36	

Genotype frequencies were compared between individuals with myopia greater than 6.00 D and less than 0.5 D. The asterisk indicates CI=confidence interval. ^#^The χ^2^ test or Fisher's exact test was performed to obtain the p value. It was considered statistically significant if p was less than 0.05.^ ##^Cp-value, p-value corrected by Bonferroni correction.

**Table 3:** Association between allelic frequencies of *CHRM1* polymorphisms and individuals with high myopia.

**Alleles**	**Individuals with myopia > 6.0D (%)**	**Individuals with myopia < −0.5D (%)**	**OR**	95% CI*	**p value^#^/Cp-value^##^**
rs11823728					
G	380 (97.9)	215 (98.6)	1		0.544/2.176
A	8 (2.0)	3 (1.4)	1.51	0.4–5.75	
rs544978					
G	336 (86.6)	166 (76.1)	1		0.001/0.004
A	52 (13.4)	52 (23.9)	0.49	0.32–0.76	
rs2186410					
A	324 (83.5)	182 (83.5)	1		0.995/3.98
G	64 (16.5)	36 (16.5)	1	0.64–1.56	
rs542269					
A	341 (87.9)	185 (84.9)	1		0.291/1.164
G	47 (12.1)	33 (15.1)	0.77	0.48–1.25	

The frequencies of alleles were compared between individuals with myopia greater than 6.00 D and less than 0.5 D. The asterisk indicates CI=confidence interval. ^#^The χ^2^ test or Fisher's exact test was performed to obtain the p value. It was considered statistically significant if p was less than 0.05. ^##^Cp-value, p value corrected by Bonferroni correction.

Moreover, we selected four haplotypes by phase software v2.1.1 (Table 4). The haplotypes distributions of the Ht1 (G/G/A/A), Ht2 (G/G/G/A), Ht3 (G/A/A/G), and Ht4 (C/A/A/A) were also compared between the two groups. The haplotype Ht4 (C/A/A/A) was significantly difference between the two groups (p=3.4×10^−5^, OR: 0.1, 95% CI: 0.03–0.34). The haplotypes frequencies of Ht1, Ht2, and Ht3 did not differ significantly between the two groups (p=0.258, 0.6, and 1.56, respectively; Table 4).

**Table 4:** Odds ratios and 95% confidence intervals for the association between *CHRM1* haplotypes and myopia.

Haplotype*	rs11823728	rs544978	rs2186410	rs542269	**Individuals with myopia > 6.0D (%)**	**Individuals with myopia < −0.5D (%)**	p value**,^#^/ Cp-value^##^	Odds Ratio (95% CI***)
Ht1	G	G	A	A	272	138	0.086/0.258	1.36 (0.96–1.93)
Ht2	G	G	G	A	61	26	0.2/0.6	1.38 (0.84–2.25)
Ht3	G	A	A	G	38	25	0.52/1.56	0.84 (0.49–1.43)

The asterisk indicates the order of polymorphisms comprising *CHRM1* haplotypes: rs11823728, rs544978, rs2186410, and rs542269. The haplotypes were identified by the Bayesian statistical method available in the program, Phase 2.1. The double asterisk means the χ^2 ^test (2×2 table) was performed to obtain the p value. The number of haplotypes in eyes with myopia greater than or equal to 6.0 D and less than or equal to 0.5 D with Ht1 was compared with the number of haplotypes in eyes with myopia greater than or equal to 6.0 D and less than or equal to 0.5 D without Ht1. A p value of less than 0.05 was considered statistically significant. Percentages may not add up to 100% because of the presence of rare haplotypes (below 0.3%) not presented in this table. ^#^The χ^2^ test or Fisher's exact test was performed to obtain the p-value. The percentages of people with and without HT1 were compared between myopia and control groups. It was considered statistically significant if p was less than 0.05. ^##^Cp-value, p-value corrected by Bonferroni correction. The triple asterisk indicates CI=confidence interval.

A logistic regression was used to determine the strength of association of the four SNPs (Table 5) with high myopia. Significant associations were found by the backward stepwise procedure for all SNPs except S3 (p=0.9219). S2 had the highest degree of association in the model (p=0.000) followed by S4 (p=0.002) and then S1 (p=0.031) (Table 5). In the test of HWE, there were departures from HWE for S1 in both the control and high myopia groups (p=0.048 and 0.023, respectively) and for S2 in the control group (p=0.012). The S3 and S4 polymorphisms were both in HWE.

**Table 5:** The main effects of SNPs by stepwise logistic regression procedure.

**Null model**	**Alternative model**	**p value**
S1+S2+S3+S4	S1+S2+S4	0.9219
S1+S2+S3+S4	S2+S3+S4	0.031
S1+S2+S3+S4	S1+S3+S4	0.000
S1+S2+S3+S4	S1+S2+S3	0.002

Four polymorphisms were selected in this study and we defined them as S1: *CHRM1* rs11823728, S2: *CHRM1* rs544978, S3:* CHRM1* rs2186410, S4: *CHRM1* rs542269.

## Discussion

Genetic studies have uncovered some polymorphisms, and separate loci are correlated with high myopia including those on chromosomes 18p and 12q [23,24] and on the genes of myocilin [25], *TGF* (transforming growth factor) [26],*PAX6* (paired box gene 6) [27], and *COL1A1* (collagen, type I, alpha 1) [26]. This indicates that there is a genetic predisposition to developing high myopia. However, no one gene can be solely responsible for the development of myopia, especially considering the wide variability in the prevalence of myopia across different ethnic groups [27-31]. The mechanisms by which myopia develops are further obscured by the uncertainty regarding the role of environmental factors. There is a higher prevalence of myopia among individuals with higher education levels than among members of the general population. Therefore, medical students were chosen as participants in the present study. Because both the high myopia and control groups constituted medical students, we believed that the bias associated with environmental influences would be minimal.

Clinical trials in school-aged patients have shown the effectiveness of daily atropine administration, which reduces the progression of myopia by approximately 60% at least in the first year of treatment [27,28]. The effect of atropine was suspected by depression of neuronal activity and leads to an increase in the general release of retinal neurotransmitters [22]. Furthermore, there is evidence that pirenzepine and himbacine inhibit myopia in a dose-dependent fashion, suggesting that these drugs mediate their effects through a receptoral mechanism [13,14]. Different muscarinic antagonists have been investigated for their individual effectiveness in reducing myopia. The efficacy of atropine, pirenzepine (M1 selective antagonist), himbacine (M4/M2 selective antagonist), and the nonselective muscarinic antagonist, oxyphenonium, were all effective in the inhibition of myopic eye growth [8,9,13,14], but individual mechanisms were still unknown. Since the results of an in vitro study suggest that the Ml mAChR subtype is most likely involved in regulating scleral growth [16], we selected it as the target gene in this study.

With stepwise logistic regression, we found an association between three of the mAChR polymorphisms (S1: *CHRM1* rs11823728, S2: *CHRM1* rs544978, and S4: *CHRM1* rs542269) and high myopia. No association was found for S3 (rs2186410). The mAChR polymorphism most strongly associated with high myopia was S2 (*CHRM1* rs544978; p=0.000) followed by S4 (*CHRM1* rs542269; p=0.002) and then S1 (*CHRM1* rs11823728; p=0.031). This was compatible with the genotype study. The frequencies of the AA genotype of S2 and the GG genotype of S4 were significantly less in the control group than in the study group, that is, they had protective effects of subjects suffering from high myopia (OR for both: 0.08; 95% CI: 0.02–0.29 and 0.02–0.36, respectively). Allele analysis revealed that the “A” allele of S2 had a significant protective effect against the development of high myopia (p=0.004; OR: 0.49; 95% CI: 0.32–0.76). However, the distribution of the “G” allele of S4 was not significantly different between the high myopia and control groups. These data indicate that the “GG” homologous genotype of S4 protects against the development of high myopia, and this function did not establish when only one “G” allele existed. Moreover, we observed that the frequencies of the haplotype Ht4 (G/A/A/A) differed significantly between the two groups (p=3.4×10^−5^). The Ht4 haplotype occurred more often in the control group than in the high myopia group (7.3% and 0.34%, respectively). The Ht4 haplotype might play a role in preventing the development of high myopia (OR: 0.1, 95% CI: 0.03–0.34). In conclusion, S1, S2, and S4 co-contributed to the genetic background of high myopia.

Our study design has strengths and limitations. We screened a large number of study subjects, and our study population was relatively homogenous in terms of ethnicity, geographic location, and age. The limitation was that the target polymorphisms detected in this study were in the 3′UTR and in the intron. These polymorphisms might contribute to the physiologic function insignificantly. 3′UTR may alter the RNA stability and/or its translational efficiency, and the intron may alter mRNA splicing. All these implications should be confirmed by experimental data. We minimized errors in genotyping and validated our findings by repeating the genotyping analysis several times and obtaining consistent results. Therefore, occurrence of genotyping errors in this study was kept to a minimum. In the HWE test, however, two of the SNPs considered in our study were not in HWE. The deviations from HWE might be a sign of mutation and may increase possible association with myopia. This disequilibrium became other evidence of the association of the polymorphisms in *CHRM1* with high myopia. Moreover, we tried to trace the ancestry background of controls and patients. Eighty-five percent are of Minnan descendants, 5% of them are Hakka descendants, and the remaining 10% are a mixed population of Minnan, Hakka, and Canton descendants. According to the paper published by Pan et al. [32], they mentioned that the SNP profiles in the major histocompatibility complex (MHC) region (6p21.3) showed no significant difference among Minnan descendants, Hakka descendants and mixed population of Minnan and Hakka descendants, which indicate the homogeneity of the population. Thus population stratification should not produce in this study [32].

In the future, further studies are needed to confirm a role of *CHRM1* in high myopia. Indeed, we intend to replicate the present study in a separate population group such as a non-student sample. We also plan to analyze the genotypes of the parents of the highly myopic participants of the current study to minimize bias. Further, we intend to analyze data across the spectrum of refractive error as obtained from all 3,000 participants screened to further understand the relationship between *CHRM1* and the severity of myopia. Complex diseases such as diabetes, cancer, asthma, and arthritis are probably caused by subtle changes in multiple genes combined with environmental and lifestyle factors. Myopia is considered a complex and multigenic disease involving several overlapping signaling pathways, each mediated by a group of distinct genetic profiles. Therefore, studying genetic polymorphisms that are related to the mechanisms of myopia can help to further clarify the relationship between genetics and myopia. The discovery of associations between myopia and various genetic markers has helped increase the knowledge needed for the prevention and treatment of myopia. As with *CHRM1*, *CHRM4* encodes a receptor important in the progression of myopia and also needs to be investigated.
